# Relevance of induced and accidental hypothermia after trauma-haemorrhage–what do we know from experimental models in pigs?

**DOI:** 10.1186/2197-425X-2-16

**Published:** 2014-05-15

**Authors:** Frank Hildebrand, Peter Radermacher, Steffen Ruchholtz, Markus Huber-Lang, Andreas Seekamp, Sascha Flohé, Martijn van Griensven, Hagen Andruszkow, Hans-Christoph Pape

**Affiliations:** Department of Orthopedic Trauma and Reconstructive Surgery and Harald Tscherne Laboratory, University of Aachen, Pauwelsstraße 30, Aachen, 52074 Germany; Department of Anaesthesiology, University of Ulm, Ulm, 89084 Germany; Department of Orthopedic Trauma, Hand- and Reconstructive Surgery, University of Marburg-Giessen, Marburg, 35043 Germany; Department of Orthopedic Trauma, Hand-, Plastic- and Reconstructive Surgery, University of Ulm, Ulm, 89084 Germany; Trauma Department, University of Schleswig-Holstein, Kiel, 24105 Germany; Department of Orthopedic Trauma and Hand Surgery, University of Düsseldorf, Duesseldorf, 40225 Germany; Department of Experimental Trauma Surgery, Klinikum rechts der Isar, Technical University Munich, Munich, 81675 Germany

**Keywords:** Multiple trauma, Haemorrhage, Accidental hypothermia, Induced hypothermia, Pigs

## Abstract

Recent experimental research has either focused on the role of accidental hypothermia as part of the lethal triad after trauma or tried to elucidate the effects of therapeutically induced hypothermia on the posttraumatic course. Induced hypothermia seems to reduce the mortality in experimental models of trauma-haemorrhage. As potential mechanisms, a decrease of cellular metabolism, beneficial effects on haemodynamic function and an attenuation of the inflammatory response have been described. However, negative side effects of hypothermia have to be considered, such as impairment of the coagulatory function and immunosuppressive effects. Furthermore, the optimal strategy for the induction of hypothermia (magnitude, duration, timing, cooling rate, etc.) and subsequent rewarming remains unclear. Nevertheless, this piece of information is essential before considering hypothermia as a treatment strategy for severely injured patients. This review aims to elaborate the differences between accidental and induced hypothermia and to summarize the current knowledge of the potential therapeutic use of induced hypothermia suggested in porcine models of trauma-haemorrhage.

**Electronic supplementary material:**

The online version of this article (doi:10.1186/2197-425X-2-16) contains supplementary material, which is available to authorized users.

## Review

### Introduction

The origin of hypothermia can differ fundamentally reflected by classifications as endogenous, controlled or accidental hypothermia. Endogenous hypothermia results either from metabolic dysfunctions with decreased heat production (e.g. hypothyroidism) or central nervous system dysfunctions with insufficient thermoregulation (e.g. tumour, trauma). Induced hypothermia, achieved by active cooling, is clinically used after cardiac arrest and in cardiac surgery for its mainly cytoprotective effects. Accidental hypothermia is defined as an unintentional decrease in core temperature during cold exposure in individuals without intrinsic thermoregulatory dysfunction [1].

A considerable number of patients presenting with accidental hypothermia are trauma victims with an incidence between 12% and 66% at arrival in the emergency room [2–4]. Clinical experience suggests that accidental hypothermia may be a major cause of posttraumatic complications, without being an independent prognostic factor for adverse outcome [5, 6]. The crucial core temperature in trauma patients seems to be approximately 34°C, and mortality rates of up to 100% have been reported in trauma patients with a core temperature <32°C [1, 7]. As pathophysiological reasoning, hypothermia induces platelet dysfunction and impairs the plasmatic coagulation system, especially below a threshold of 33°C. This hypothermia-induced coagulopathy was shown to be associated with significantly increased blood loss [8]. Therefore, the reversal of the ‘lethal triad’ of acidosis, coagulopathy and accidental hypothermia is indispensable for the successful treatment of bleeding trauma patients.

The deleterious effects of accidental hypothermia in multiple trauma patients contrast the beneficial effect of induced hypothermia on organ function during ischemia in elective surgery [9]. Furthermore, experimental studies have highlighted the protective effects of induced hypothermia in the setting of haemorrhagic shock with less pronounced organ dysfunction and reduced histological scores of organ damage, especially when induced rapidly after the traumatic insult [10, 11]. The beneficial effects of induced hypothermia might be explained by ameliorating or preventing the initiation and progression of an overwhelming systemic inflammatory response (‘systemic inflammatory response syndrome’, SIRS) [9–11]. Others have suggested that induction of hypothermia results in a reduction of oxygen demand while maintaining relative oxygen delivery and thereby maintaining levels of energy-rich phosphates, which is advantageous during ischemic periods by prolonging the golden hour of shock [12]. In contrast, accidental hypothermia is associated with reduced concentrations of energy-rich phosphates and higher lactate levels as a result of insufficient tissue perfusion and failure of the organism to maintain normal body temperature. Therefore, accidental hypothermia seems to be a result and driver of severe shock finally leading to adverse outcome [13]. In conclusion, it seems to be essential to differentiate between induced and accidental hypothermia, as these different entities clearly exert divergent effects on the inflammatory response and posttraumatic organ function.

Recent experimental research has tried to define the role of hypothermia as part of the lethal triad after trauma as well as to balance out potentially beneficial versus adverse aspects of therapeutically induced hypothermia on the posttraumatic course. Furthermore, promising treatment protocols for the induction of hypothermia have been optimized (optimal magnitude, duration, timing, cooling method and cooling or rewarming) and evaluated [14–29].

This review aims to differentiate between these two entities of hypothermia and to summarize the current knowledge of the potential therapeutic use of induced hypothermia in the posttraumatic setting. We set the focus on evolutionary highly developed, large-sized species (pigs) as, e.g., the size of the organism (small vs. large animal models) significantly affects the reaction on therapeutically induced hypothermia. Small animals were shown to be very sensitive to hypothermia due to their high surface-to-volume ratio, which has to be taken into account when comparing various studies in this field [30]. Furthermore, small animals present with ‘non-shivering thermogenesis’ [31]. Therefore, porcine models were chosen because their physiological response to trauma-haemorrhage simulates the human response more closely than any other non-primate species. Except for some minor differences, pigs exhibit cardiovascular, haematological, immunological and electrolyte profiles that are almost identical to those in humans [32]. Only the coagulation system differs significantly to humans, with pigs being naturally in a hypercoagulatory state.

### Methods

The following criteria were used to determine eligibility of a study to be included in this review. Inclusion criteria were isolated or combined trauma-haemorrhage models, use of pigs as experimental animals, English or German language and the use accidental or induced hypothermia. The use of other large animals or small animals resulted in exclusion of the study.

A literature search was carried out on Medline, Embase and Cochrane for studies published until July 2013 on the topic of the effects of accidental and induced hypothermia in porcine models of isolated or combined trauma-haemorrhage. The following key words were used: ‘accidental hypothermia’, ‘spontaneous hypothermia’, ‘induced hypothermia’, ‘therapeutic hypothermia’, ‘pigs’, ‘swine’, ‘trauma’, ‘injury’, ‘hemorrhage’, ‘fracture’ and ‘bleeding’. The references of selected studies were also perused for articles that may have been missed via the electronic search.

#### Study selection

The title and abstract of all identified studies were examined by one reviewer (F.H.). Then, the entire article was obtained and assessed for suitability by two of the authors (F.H., S.F). Any issue pertaining to eligibility of studies was solved via discussion with the senior author (H-C.P.). Data were extracted according to the information presented in Additional file 1: Table S1 and Additional file 2: Table S2.

### Accidental hypothermia as part of the lethal triad

Beside acidosis and coagulopathy, accidental hypothermia is recognised as one main pillar of the lethal triad after severe trauma [19, 33]. Both acidosis and hypothermia are well known to exert significant effects on the coagulation system, resulting in a significantly higher blood loss due to an increased bleeding time [34] and enhanced mortality rate [34, 35]. Acidosis was proposed to be more detrimental than hypothermia in the development of coagulopathy [34]. However, hypothermia also revealed significant effects on the coagulation system with decreased fibrinogen concentrations (without affecting fibrinolysis) and impaired thrombin generation. In this regard, the onset of thrombin generation is remarkably delayed at a body temperature of 32°C [34]. Hypothermia also induces temporary thrombocytopenia (28% drop at 32°C) and platelet dysfunction [36, 37]. Below a threshold of 33°C, a decrease of body temperature additionally interferes with the plasmatic coagulation system by decreasing the activity of the associated enzymatic reactions [10, 36]. In fact, Martini et al. demonstrated that hypothermia and haemorrhage affected different aspects of the plasmatic coagulation process while both caused coagulation abnormalities: induction of hypothermia (32°C) alone did not result in significant changes in prothrombin time (PT) or activated partial thromboplastin time (aPTT) (assayed at 32°C). In contrast, hypothermia in combination with haemorrhagic shock (loss of 35% of total blood volume) with subsequent haemodilution (by resuscitation with Ringer's solution) led to a prolongation of PT by 40% (assayed at 32°C), without significant effects on aPTT [36]. It was concluded that hypothermia might have differential effects on the intrinsic and extrinsic pathways of the coagulation cascade. The effects of trauma and hypothermia on PT were confirmed by other studies [34, 37]. In contrast to aPTT, the activated clotting time (ACT) was prolonged by either isolated hypothermia or hypothermia in combination with haemorrhage and resuscitation [36]. As ACT is similar to aPTT except that it includes the interaction of platelets with other clotting components, the observed differences between changes of ACT and aPTT suggest that platelets are an important contributor to the hypothermia-induced effects on coagulation [36].

Using thrombelastography, hypothermia prolonged the initial clotting time and clot formation time and decreased clotting rapidity without affecting clot strength [36, 37]. In contrast, moderate haemorrhage and resuscitation alone had no effect on the initial clot formation or clot rapidity, but resulted in impaired clot strength. Thus, hypothermia and haemorrhage seem to have different effects on the coagulation function. Martini et al. concluded from their studies that the functional changes of the coagulation cascade are due to platelet dysfunction from hypothermia and depletion of fibrinogen and platelets from haemorrhage and resuscitation. When hypothermia and haemorrhage were combined, all abovementioned parameters were impaired [34, 36].

As hypothermia-induced coagulopathy cannot be reversed with administration of clotting factors, but can be easily corrected when hypothermia is corrected [10], passive or active rewarming techniques seem to be of major relevance. Both methods can be applied in an external or internal fashion. Besides warmed-air blankets or warmed fluids, the most efficient means of rewarming is extracorporeal blood warming by different kinds of pumps (e.g. continuous arteriovenous rewarming, centrifugal vortex blood pump) [19]. In a study of Garraway et al., fast rewarming from 29°C to 37°C (within 85 min versus 217 min) appeared to correct the coagulopathy sooner and consequently may reduce the risk of ongoing bleeding [19].

In conclusion, to the best of our knowledge no studies have investigated the specific effects of isolated accidental hypothermia on haemostasis. At least in part, hypothermia has been induced using cooling blankets, immersion in cool water or evaporation (Additional file 1: Table S1). Therefore, the isolated effects of accidental hypothermia as part of the lethal triad are essentially uncharacterized by previous models [38]. While induced hypothermia might be used to propose hypotheses on possible mechanism of the effects on coagulation, the definite major underlying pathomechanisms can only be assessed if hypothermia occurs spontaneously after trauma-haemorrhage, e.g. in the context of systemic hypoperfusion.

### Therapeutically induced hypothermia

#### Considerations on model design and strategies for induction of hypothermia

In contrast to elective surgery (e.g. transplantation, neurosurgery, cardiac surgery), induced hypothermia can only be deliberated after the onset of the insult in the setting of severe tissue injuries. Due to the potentially negative side effects of therapeutically induced hypothermia, the impact of a decreased body temperature was investigated in porcine trauma models in order to transfer the results to the clinical setting of multiple trauma patients. The design of these experimental studies differs in diverse aspects (e.g. kind of induced trauma, technique, timing of induced hypothermia and rewarming phase), which can be explained by the different purposes of the study or varying experimental questions.

##### Injury pattern

In general, the effects of induced hypothermia have been investigated in models with isolated haemorrhagic shock or in combined models with additional trauma load (Additional file 2: Table S2). Although volume- and pressure-controlled haemorrhage models offer a high level of standardization, the absence of uncontrolled sources of bleeding (e.g. solid organ injuries) has been identified as a limitation of studies with controlled haemorrhagic shock in which no ongoing bleeding is observed. Furthermore, a model of haemorrhage without significant additional tissue trauma is unlikely to validly predict the response to therapy in any clinical setting, as tissue injury alters the response to haemorrhage.

The implementation of solid organ injuries seems to be of particular importance in studies investigating the potential clinical application of induced hypothermia after trauma-haemorrhage in order to observe whether bleeding control is worsened when lowering the body temperature [11, 39]. Therefore, models with additional liver, lung or soft tissue trauma (with and without additional fractures) have been developed which allowed investigating whether the injured locus starts to re-bleed after the induction of hypothermia [39, 40].

##### Time point of hypothermia induction

In diverse experimental studies, hypothermia was often therapeutically induced in parallel [41], a few minutes after [10] or even before [42, 43] the induction of haemorrhage. Although this strategy seems to be important for mechanistic considerations of hypothermia treatment, it is clearly not compatible to the clinical situation due to several reasons (Additional file 1: Table S1). Firstly, the induction of hypothermia before or in parallel to trauma-haemorrhage is impossible in the setting of multiple trauma. Secondly, a significant number of multiple trauma patients develop accidental hypothermia in the course of trauma-haemorrhage. Therefore, no hypothermia can additionally be induced under these conditions. Lastly, it might be advantageous to induce hypothermia after surgical bleeding control due to the potential coagulopathic effects, particularly in the case of additional organ injuries. Therefore, therapeutic hypothermia was induced after emergency surgery and correction of accidental hypothermia (analogous to the time point of treatment on the intensive care unit) in some combined trauma models [39, 40].

##### Velocity and technique of hypothermia induction

Numerous studies emphasized that therapeutically induced hypothermia seems to be most effective when induced rapidly after trauma-haemorrhage [10, 11, 41, 44–46]. In this context, surface cooling (evaporation cooling and ice packs), which failed to sufficiently decrease core temperatures, even had adverse effect on the posttraumatic course [47], whereas rapid induction of hypothermia by extracorporeal shunt circulation prolonged short-term survival in the same model. However, it was also supposed that a continuous and prolonged attempt to reduce the core temperature may add additional stress and reduced the survival rate [41].

Besides the rapidity of reducing the body temperature, other factors have also been claimed to have a significant physiological impact on the posttraumatic course after induced hypothermia [48]. It has been suggested that hypothermia should be induced under anaesthesia and muscle relaxation in order to suspend the energy-consuming shivering and the stress response of the sympathetic nervous system to hypothermia [48]. Furthermore, the technique of hypothermia induction seems to be decisive. In this regard, different authors reported that the infusion of 4°C lactated Ringer's solution prolongs survival [44, 45, 48]. However, induction of hypothermia with infusion of saline at room temperature and surface cooling was found to be more effective after haemorrhagic shock [48]. As a possible mechanism, a faster cold response with vasoconstriction and decreased tissue perfusion was suggested, which might have resulted in a worse outcome [48]. In many studies, a rapid decrease of core temperature after severe trauma was reached by extracorporeal cooling while, noteworthy, no adverse effects of fast cooling were reported [10, 39, 40, 43]. For example, some studies used a heparin-free roller pump that has been described to reduce body temperature in a reliable fashion [39, 40]. Others used cardiopulmonary bypass techniques (with heparin) to induce hypothermia, with a rather rapid cooling rate of 2°C/min [10].

##### Rewarming

Rewarming was often performed within the resuscitation process or shortly thereafter (Additional file 1: Table S1). It has been shown that long-term survival after induction of therapeutic hypothermia is also influenced by the rate of rewarming [46]. In this context, a rewarming rate of 0.5°C/h has been proven to be most effective, whereas a faster increase of body temperature (>1.0°C/h) did not significantly alter survival after induced hypothermia compared to normothermic animals in a haemorrhagic shock model [46]. However, rewarming after induced hypothermia should always be evaluated in the context with the duration of hypothermia as rebound effects after too rapid rewarming might depend on the time period between the trauma impact and the rewarming phase.

#### Effects of induced hypothermia on the posttraumatic course

##### Survival and haemodynamics

Various experimental studies demonstrated that therapeutic induction of hypothermia can significantly improve posttraumatic survival [10, 41, 44, 45, 48]. This beneficial effect of a decreased body temperature has been shown for different degrees of hypothermia. Wu et al. found that mild hypothermia (34°C) is effective in a model of haemorrhagic shock that includes resuscitation and intensive care treatment [48]. In another study [49], the induction of moderate hypothermia of 30°C resulted in a reduction of posttraumatic mortality. In a study of Alam et al., the protective effects of profound induced hypothermia (10°C) was highlighted [11], which were confirmed in a model of uncontrolled haemorrhage with major vascular, solid organ and hollow viscus injuries [50]. Furthermore, it seems to be interesting that profound hypothermia (10°C) was found to be more effective than ultraprofound hypothermia of 5°C [51]. It is also to be considered that the duration of hypothermia seems to have an additional impact on the beneficial effects of hypothermia after uncontrolled haemorrhage - at least for profound hypothermia. In this context, 60 min of hypothermic arrest seems to be the upper limit, as a significant decrease in survival as well as an increase of postoperative complications has been described after 120 min [52].

Focusing on the association between the technique of hypothermia induction and haemodynamic function as well as survival, it was shown that the administration of cold crystalloid solution (4°C) prolongs survival after uncontrolled haemorrhage [44], which coincided with a lower heart rate, decreased myocardial oxygen requirements and an improved stroke volume index during haemorrhagic shock, probably leading to a better preservation of cardiac function [44]. Wu et al. reported similar findings with best results for induction of hypothermia with infusion of saline at room temperature and surface cooling. In contrast, rapid cooling ice-cold fluids showed a decreased survival rate in the same model. As the infusion of ice-cold fluid was associated with increased mean arterial pressure (MAP) and lactate levels, the authors assumed that vasoconstriction with decreased tissue perfusion played a role in worsening the outcome [48]. The authors postulated that rapid changes in core temperature might trigger a faster cold response with potentially detrimental vasoconstriction and decreased tissue perfusion. Therefore, the authors concluded that it is important to suspend the shivering and the response of the sympathetic nervous system to hypothermia by using anaesthetics and muscle relaxants at the time of hypothermia induction [48].

External cooling by a ‘hypothermic bed’ decreased MAP, cardiac output and heart rate after isolated haemorrhagic shock [30, 49, 53]. The cardiovascular adaption to hypothermia after haemorrhage obviously consists of an early heart rate-induced reduction of cardiac output and a later decrease of MAP [30]. Under primary limited (SAP >80 mmHg) and subsequent full (SAP >90 mmHg) resuscitation and deep anaesthesia, surface cooling by means of ice packs to 33°C (0.5°C to 1°C/h) resulted in a significantly improved survival rate compared to normothermia [45] in an isolated haemorrhagic shock model. In contrast, the same group only observed a trend towards improved survival and a transient elevation of markers of organ function after combined trauma and hypothermia induction to 34.5°C. Possible explanations may be the shorter hypothermic period and the milder degree of hypothermia in the latter study [42].

Besides the technique, degree and duration of induced hypothermia, the velocity of decreasing body temperature has a significant impact as well. Takasu et al. found that under light anaesthesia, surface cooling (evaporation cooling and ice packs) during severe haemorrhagic shock without any fluid resuscitation failed to decrease core temperature and even shortened the survival time in pigs. Moreover, animals in the surface cooling group exhibited a more pronounced decline of MAP, arterial pH and cardiac index than the normothermic group [47]. It was supposed that a continual and prolonged attempt to reduce the core temperature added additional stress and reduced the survival rate [41]. However, rapid extracorporeal cooling to 34.5°C resulted in a significantly increased survival with higher MAP and stroke volume index as well as a lower heart rate compared to normothermic animals in the same model [41]. Accordingly, Alam et al. described hypothermia to be most effective if rapidly induced with a rate of 2°C/min (compared to 1°C or 0.5°C).

In summary, the vast majority of experimental studies support a rapid decrease of core temperature as the strategy of choice for hypothermic treatment after severe trauma [10, 41, 43, 44, 51, 54]. Furthermore, the duration and degree of induced hypothermia as well as the deepness of anaesthesia and muscle relaxation seem to have an impact on the therapeutic effects of hypothermia.

##### Coagulation system

Hypothermia is well known to modify the cellular and plasmatic components of the coagulation system. However, in contrast to the coagulopathic effects of the lethal triad, diverse experimental studies failed to find significant differences in signs of coagulopathy (PTT, platelet count, INR, TEG) between normothermic pigs and after induction of hypothermia (between 33°C and 35°C) [42, 43, 45]. In accordance, therapeutic induction of profound total body hypothermia (10°C) for approximately 60 min as well as induced mild hypothermia (34°C) for about 12 h did not result in a measurable increase of postoperative bleeding in a combined trauma model [10]. Also in other studies, therapeutically induced hypothermia did not result in an enhanced incidence of bleeding complications compared to normothermic animals [44, 48, 49, 53]. This might be explained by the fact that therapeutically induced hypothermia does not seem to affect maximum clot strength [40]. As the maintenance of clot strength has been shown to be crucial for transfusion requirements [36], it is tempting to speculate that the effects of therapeutic hypothermia on the coagulation system are rather limited - at least in porcine models of trauma-haemorrhage [40]. However, the coagulopathic effects of induced hypothermia are discussed controversially at least for body temperatures below 33°C. In this regard, Gröger et al. found a prolonged clotting time and reduced clot firmness at 32°C, whereas 35°C had no effects on these parameters [43]. Furthermore, relevant differences in the coagulation system between pigs and humans have to be considered, when interpreting the effects of hypothermia on coagulation. In pigs, a general hypercoagulatory situation has been described compared to the human situation. Furthermore, it has so far not been possible to induce trauma-haemorrhage-related coagulopathy in pigs [55, 56]. Therefore, further experimental studies (e.g. in other animal species or in pigs with a different genetic background) are needed to define a safe therapeutic window in terms of degree and duration before induced hypothermia might be applicable in the clinical setting. Overall, the secondary induction of hypothermia after primary surgical bleeding control and therapy of trauma-related coagulopathy on the intensive care unit might represent a treatment strategy which can minimize the bleeding risks associated with a therapeutic decrease of body temperature [40].

##### Metabolism

The hypothermia-associated decrease of the metabolic rate in key organs is supposed to be one of the most protective strategies for cellular function during periods of haemorrhage, ischemia and reperfusion. It is well established that alterations in temperature influence biologic reactions with a 50% reduction in metabolism for every 10°C decrease in body temperature (i.e. ‘Q10’ effect) [10]. In addition, a significant reduction in blood catecholamine levels after the induction of hypothermia might additionally diminish the metabolic rate after trauma-haemorrhage [49, 53]. These decreased metabolic and oxygen demands under hypothermic conditions have been associated with reduced oxygen extraction ratio and increased pO_2_ values [49, 53] as well as with improved oxygen delivery indices in mechanically ventilated pigs [45]. Furthermore, this increased oxygen supply might be beneficial as it counteracts the development of an anaerobic metabolism after trauma-haemorrhage, thereby preserving energy-rich phosphate (e.g. ATP) levels and attenuating a tissue energy depth. Accordingly, decreased posttraumatic lactate levels after induction of hypothermia have been observed [43, 45, 49]. It was also assumed that the maintenance of ATP stores might contribute to a switch of cell death from necrosis to apoptosis, as apoptosis has been associated with higher ATP content compared to necrotic cell death. In accordance, a more pronounced apoptosis after therapeutic induction of hypothermia was observed, whereas cell necrosis was attenuated [43]. This might lead to less local and systemic inflammation as apoptotic events canonically cause much less inflammation in comparison to necrotic events.

The effects of hypothermia on the development of metabolic acidosis after trauma-haemorrhage have been controversially discussed in the literature. In a study by Gröger et al. [43], induction of hypothermia resulted in metabolic acidosis which persisted even after rewarming until the end of the experiment. The development of this metabolic acidosis was at least partly explained by an enhanced fat metabolism with increased free fatty acid concentrations and reduced carbohydrate oxidation. A decreased hepatic clearance of acid metabolites under hypothermic conditions might also contribute to this effect. However, others [53] described no effects of moderate hypothermia (30°C) on blood pH and base excess after isolated haemorrhagic shock. Again, the time point of hypothermia induction as well as duration and severity of induced hypothermia might represent significant factors for these differences.

Besides the effects on metabolism, some studies [43, 49, 53] also found an impact of hypothermia on electrolyte levels. Although a decreased body temperature has been described to alter the function of the sodium-potassium pump resulting in increased potassium levels, induced hypothermia after trauma-haemorrhage maintained normokalemia. In contrast, normothermia was associated with hyperkalemia. This beneficial aspect of hypothermia was explained by a limitation of tissue damage by induced hypothermia. It seems interesting in this study that the higher serum levels of creatine kinase (CK) in hypothermic animals were not a result of muscular tissue destruction but of shivering, as previous blocking of all muscle activity by drugs prevented this CK increase [53].

##### Inflammatory response

The modulation of the inflammatory response after trauma-haemorrhage might be another mechanism by which induced hypothermia exerts protective effects during the posttraumatic course. Induced hypothermia has been shown to attenuate or at least delay the pro-inflammatory and oxidative stress response which was associated with a transitory reduction of kidney and liver dysfunction as well as reduced histological damage (e.g. reduced infiltration of polymorphonuclear granulocytes) in these organs [43, 50, 53]. The augmented liberation of protective heat shock proteins might further contribute to the beneficial effects of hypothermia under those conditions [50].

The hypothermia-associated alteration of the T cell cytokine production pattern with a conversion from a Th-1 to a Th-2 cytokine pattern has been shown to result in an anti-inflammatory immunosuppressive profile [1]. It was speculated that hypothermia and trauma might activate the hypothalamic-pituitary-adrenocortical (HPA)-axis, resulting in an increased glucocorticoid secretion. Furthermore, it has been shown that hypothermia preserves plasma glucocorticoid concentration. As glucocorticoid is supposed to be a strong inducer of IL-10 production, these effects of hypothermia might contribute to the hypothermia-associated increase of IL-10 levels. It was therefore suggested that the immunosuppressive profile as well as hypothermia-associated vasoconstriction and local tissue hypoxia may increase the risk of infections and worsen outcome [1, 57]. In accordance, Alam et al. observed an increase in infectious complications after a 2-h period of profound hypothermia (10°C) [52]. However, such effects on the incidence of infectious complications were not described for a duration of 60 min [10, 52], even if an additional colon injury was induced. Therefore, it has to be assumed that the impact of hypothermia on immune function is to some extent time-dependent [10]. Furthermore, for the long-term effects of hypothermia on the pro-inflammatory response, it might be speculated that hypothermia results in a delayed or prolonged release of inflammatory mediators. In this context, Gröger et al. found the highest TNF-α, IL-6 and 8-isoprostane blood levels in hypothermic animals (32°C) at 12 h after resuscitation and at the end of the study period, whereas induced hypothermia of 35°C resulted in an almost similar response as normothermia [43].

### Limitations

Besides the well-described variables which have some influence on the results of experimental studies (anaesthesia before trauma, application of drugs and infusions, small sample sizes) [58], the species-specific differences between humans and pigs deserve particular attention.

It is well known that it is difficult to achieve a state of coagulopathy in pigs [23]. In fact, induction of haemorrhagic shock, chest and abdominal trauma did not result in a significant reduction of coagulation activity [39]. This is in line with results of previous studies in which moderate haemorrhage alone and resuscitation with lactated Ringer's solution did not suffice to deplete coagulation substances to alter clotting time [23, 36]. Other authors performed haemodilution before the induction of injury in order to standardize coagulopathy, which certainly fails to mimic the clinical situation [59]. Therefore, data on the effects of therapeutically induced hypothermia on posttraumatic coagulation disorders originating from porcine models and the transferability to the human situation have to be interpreted carefully [23, 36, 38, 44, 58, 60]. In addition, different vasopressin receptors exist in pigs and humans that may result in a different haemodynamic response to exogenously administered vasopressin [61, 62]. Porcine granulocytes should not be considered representative for the human setting due to differences of elastase release and activity. Furthermore, the reticuloendothelial system in swine is located in the pulmonary region, which is in contrast to the situation in humans. This might have significant effects, e.g., on the pulmonary artery pressure in specific situations: acute challenges in swine often result in marked pulmonary arterial hypertension [63].

Furthermore, the observation time has to be considered when interpreting the effects of hypothermia found in porcine models of trauma-haemorrhage. In many of the studies, timing was selected to mirror the clinical setting as faithfully as possible, focusing on the first golden hour after trauma [30, 44, 47, 53]. Therefore, it also has to be assumed that a limited observation time might lead to undetected treatment effects (e.g. functional recovery of endothelial or organ function) or compensatory mechanisms of therapeutically induced hypothermia. Furthermore, the incidence of re-bleeding has to be investigated due to the potential negative side effects on the coagulation system. Moreover, immunosuppressive long-term effects with an associated increased incidence of infectious complications have to be taken in account. Finally, potential rebound effects need to be considered. A delayed overwhelming inflammatory response might be postulated after termination of induced hypothermia and rewarming. Accordingly, it might be speculated that a hypothermia-associated improvement of cellular and organ function might deteriorate after rewarming and ultimately result in the same severity of cellular and organ dysfunction as maintenance of normothermia. Hypothermia also seems to affect posttraumatic apoptosis including the inhibition of activation of caspase enzymes and the preservation of mitochondrial function [64]. As the apoptotic process has been described to occur not only early but also relatively late following trauma-haemorrhage and seems to continue for up to 3 days, long-term models are of major importance before implementing therapeutically induced hypothermia in the clinical setting. In order to achieve a longer observation period, animals were extubated after 6 to 24 h in some other experiments and then observed for several days in an awake state [42, 45, 54]. Although this allows the observation of some of the long-term effects of therapeutically induced hypothermia, it is not directly comparable to the clinical situation of multiply injured patients, who are intubated, sedated and treated on the intensive care unit for several days.

However, the performance of long-term experiments also has major challenges and possible limitations. These include logistic necessities and the associated costs. Furthermore, both personnel with clinical and scientific experience in intensive care medicine and trauma surgery as well as adequately equipped facilities are needed to ensure reliable results. It also has to be taken into account that growth might be an important issue in swine when observing long-term outcomes with observation periods of several weeks. However, this aspect might be of minor importance in models with a study period of several days. It will also gain increasing importance to include potential co-morbidities in the experimental models to resemble the higher number of older trauma patients with a relevant incidence of pre-existing morbidities (e.g. arteriosclerosis, hypertension, diabetes, etc.).

## Conclusion

Overall, it remains unclear whether therapeutically induced hypothermia has a promising potential for clinical use in the posttraumatic setting. This presentation of porcine models shows a significant variability of the experimental settings. This is due to the different purposes or the aims of the studies, which result in significant differences of the conditions for induction of trauma (severity and duration of haemorrhage, isolated or combined insults) and hypothermia/rewarming (method, time points, and duration) between porcine models of trauma-haemorrhage. This might partly lead to conflicting study results. However, it is also difficult to prioritize between the significance of the studies as every model has its strengths and weaknesses without an intrinsic value of an experimental model *per se*.

In conclusion, the current state of knowledge of therapeutically induced hypothermia after severe trauma is clearly not sufficient enough to start application in the clinical setting. Induction of hypothermia as a treatment strategy for severely injured patients would require more essential information, such as the optimal rate, time point, and duration of hypothermia induction as well as of rewarming and long-term observational studies.

## Electronic supplementary material

Additional file 1: Table S1: Accidental hypothermia [14–29, 33, 35–37, 55, 56, 60, 65–69]. (DOCX 206 KB)

Additional file 2: Table S2: Induced hypothermia [10, 11, 30, 40, 45, 47–50]. (DOCX 152 KB)
