# Supplementary material for: Relevance of induced and accidental hypothermia after trauma-haemorrhage–what do we know from experimental models in pigs?
Source: Intensive Care Med Exp. 2014 May 15;2:16. doi: 10.1186/2197-425X-2-16 (PMC4512998; doi:10.1186/2197-425X-2-16)
Supplement: Supplementary file 1 — Additional file 1: Table S1: Accidental hypothermia [14–29, 33, 35–37, 55, 56, 60, 65–69]. (DOCX 206 KB) [file 40635_2013_17_MOESM1_ESM.docx]

| Author | Endpoints | Number of animals | Insult | Time to resuscitation | Blood loss | Surgical procedure | Resuscitation | Mortality | Hypo-thermia | Hypothermia induction/  rewarming | Study period/  hypothermia period | Thesis |
| --- | --- | --- | --- | --- | --- | --- | --- | --- | --- | --- | --- | --- |
| Inaba K et al. 2011 ([23](#_ENREF_23)) | - Blood loss - Mortality | - Gauze control: n=11 - Celox n=11 - QuickClot: n=11 | - Controlled hemorrhage via venous line - 10 cm hepatic injury (grade IV) with scalpel blade | After controlled hemorrhage: 0 min  After hepatic injury:  ≈12 min | Controlled hemorrhage: 35% (≈25 ml/kg BW) of total blood volume  Hepatic injury:  Gauze Control: 8.3ml/kg BW  Celox: 3.7 ml/kg BW  QuickClot: 4.6 ml/kg BW | Laparotomy and liver packing | After controlled hemorrhage:  Lactated Ringer´s three times the shed blood volume  After hepatic injury: Lactated Ringer´s to maintain a MAP of >60mmHg | 0% | 35°C | Intraperitoneal cool packs before hepatic injury/ rewarming with warm infusions | total:  2 hours intubated, 48 hours awake hypothermia  2 hours | Hypothermia as part of the lethal triad- effects of different local hemostatics |
| Garraway N et al. 2007 ([19](#_ENREF_19)) | - Rewarming time | - Conventional rewarming: n=7 - CVBP rewarming: n=8 | - Controlled hemorrhage via venous line - Splenectomy | ≈ 40 min | MAP 35-40mmHg  (≈ 35% of total blood volume) | Laparotomy | Achieve/maintain MAP of 80mmHg | 6.3% | 29°C | Intraperitoneal and external cool packs after hem. before splenectomy/ rewarming with warm intraperitoneal lavage, warm infusions, warming blanket with or without warming veno-venous pump | total:  5 hours hypothermia  Time to perform splenectomy | Hypothermia as part of the lethal triad- effects of different re-warming techniques |
| Dickneite G et al. 2008 ([16](#_ENREF_16)) | - Coagulation profile | - N=45 | - Induction of normotensive dilutional coagulopathy | Reinfusion 10 min after withdrawal | Withdrawal of whole blood and reinfusion of resuspended erythrocytes and HES | - Splenectomy - 3mm hole in the femoral neck | Reinfusion of resuspended erythrocytes and HES | 0% | 36°C | No intentional induction of hypothermia, due to infusions/ no rewarming | total:  4 hours hypothermia | Hypothermia as part of the lethal triad |
| Martini WZ et al. 2008 ([37](#_ENREF_37)) | - Coagulation profile | - n=24 | - Controlled hemorrhage via arterial line | 30 min | ≈30% of total blood volume | None | 3x the shed blood volume of lactated Ringer´s solution | Hypothermia and normothermia: 0% | 32°C | After starting hemorrhage with cold blanket/ no rewarming | total:  4 hours hypothermia  ≈4 hours | Hypothermia as part of the lethal triad- effects on coagulation |
| Martinowitz U et al. 2001 ([27](#_ENREF_27)) | - Blood loss - Clotting factor activity - Mortaity | - With FVIIa application: n=5 - Without FVIIa application: n=5 | - Induction of isovolemic, exchange transfusion - Grade V liver injury | 5,5 min after liver injury | - With FVIIa application:   ≈ 530ml   - Without FVIIa application:   ≈ 980ml | Laparotomy with splenectomy | - Isovolemic exchange transfusion:   60% of total blood volume  exchanged with HES   - After liver injury:   Return to and maintenance of preinjury MAP by infusion of lactated Ringer´s solution | 0% | 33°C | Before liver injury by cold infusion and intraperitoneal lavage/ partly rewarming with 40°C Ringers lactate during resuscitation | total:  1 hour  hypothermia  not available | Hypothermia as part of the lethal triad- effects on coagulation and rFVIIa |
| Spoerke N et al. 2009 ([60](#_ENREF_60)) | - Blood loss - Clotting factor activity - Mortality | - FFP: n=8 - LP: n=8 - 1:1 FFP-RBC: n=8 - 1:1 LP-PBC: n=8 | - Femur fracture with captive bolt gun - Controlled hemorrhage - Grade V liver injury with uncontrolled bleeding | - 30 min after con-trolled hem-orrhage - 30 sec of uncon-trolled bleeding | - Controlled blee - ding:   60% of total  blood volume   - Total blood loss:   FFP: ≈564ml  LP: ≈630ml  1:1 FFP-RBC:  ≈609ml  1:1 LP-PBC:  ≈447ml | Laparotomy | - After controlled bleeding:   3 times the controlled  hemorrhage volume with  0.9% saline solution   - After uncontrolled bleeding:   Either FFP, LP, FFP-PRBC in 1:1 ratio or LP-PRBC in 1:1 ratio with infusions equal to the blood removed during controlled hemorrhage | 0% | 33°C | After femoral fx and laparotomy, before hemorrhage by intraperitoneal lavage/ no rewarming | total:  4 hours hypothermia | Hypothermia as part of the lethal triad- Effects of FFP and lyophilized plasma |
| Cho SD et al. 2009 and Lee TH et al. Transfusion 2013 ([15](#_ENREF_15), [26](#_ENREF_26)) | Cho SD 2009:   - Development of new model   Lee TH 2013:   - Coagulation profile - Inflammatory response | Cho SD 2009:   - n=29 (+8 deceased animals)   Lee TH 2013:   - n=48 | - Femoral fracture by captive bolt gun - Controlled hemorrhage - Grade V liver injury | - Controlled hemorrhage   30 min   - Uncontr. hemorrhage 30 sec | - Controlled hemorrhage:   60% of total  blood volume | Laparotomy | - If MAP <25mmHg during blood withdrawal:   165ml/min of 0.9% saline solution   - After shock (controlled hemorrhage) period:   Hemorrhage volume in a 3:1 ratio of 0.9% saline solution | n.a. | 33°C | After hemorrhage induction by intraperitoneal lavage/no rewarming | total:  2 hours hypothermia  ≈ 100 min | Hypothermia as part of the lethal triad- standardization of a new porcine model |
| Alam HB et al. 2011 ([14](#_ENREF_14)) | - Mortality - Organ function | - Hextend (6%)   n=8   - Fresh whole blood   n=7   - Hyperoncotic plasma   n=6   - Valproic acid+ hextend   n=6 | - Rib fracture (left seventh) with soft tissue trauma - Controlled hemorrhage - Grade IV liver injury and grade V spleen injury with uncontrolled hemorrhage | - Controlled hemorrhage   30 min   - Uncontrolled hemorrhage   2.5-6 min | - Controlled hemorrhage:   50% of total  blood volume | Laparotomy | - If MAP <30mmHg during blood withdrawal:   165ml/min of 0.9% saline solution   - After shock (controlled hemorrghe) period:   Hemorrhage volume in a 3:1  ratio of 0.9% saline solution   - After shock (uncontrolled hemorrghe) period:   a) Hextend (6%)  b) Fresh whole blood  c) Hyperoncotic plasma or  d) Valproic acid+hextend  ⇒ total volume equal to blood volume removed during controlled hemorrhage | - Hextend (6%)   75%   - Fresh whole blood   0%   - Hyperoncotic plasma   17%   - Valproic acid+ hextend   50% | 33°C | Before liver and spleen injury by intraperitoneal lavage/ rewarming with warming blanket | total:  4 hours intubated, 7 days awake hypothermia  ≈ 90 min | Hypothermia as part of the lethal triad- effects of spray dried plasma |
| Honickel M et al. 2011 ([22](#_ENREF_22)) | - Clotting factor activity - Hepatic injury | - n=14 | - Induction of isovolemic, exchange transfusion + liver injury with 100-150N, 200-300N or 300-400N) | 5 minutes | 100-150N:  239ml  200-300N:  23ml/min  300-400N:  68ml/min | Laparotomy with splenectomy | Splenectomy:  Ringer´s lactated solution 3x the weight of the spleen  Isovolemic exchange transfusion:  80% of total blood volume exchanged with HES (max. 50ml/kg BW) and Ringer´s solution (ratio: 1:1.2-1.5), retransfusion of processed red blood cells  Liver injury:  125 ml/min for 8 minutes, thereafter 25ml/kg BW/h | 200-300N:  100% (survival time: 76 min)  300-400N:  100% (survival time: 30 min) | 33°C | Infusion of crystalloids before trauma induction/ no rewarming | Total  Max. 2 hours hypothermia  Max. 2 hours | Hypothermia as part of the lethal triad- effects on coagulation |
| Shuja F et al. 2008 and 2011 ([33](#_ENREF_33), [65](#_ENREF_65)) | Shula 2008 +2011   - Clotting factor activity - Mortality | Shuja 2008   - FFP. N=10 - FDP=10   Shuja 2011   - Spray-dried plasma: n=5 - FFP: n=5 - FDP: n=5 | - Femoral Fx by captive bolt gun - Volume-contr. hem. via arterial cath. - Uncontr. hem. (grade V liver injury) | Controlled hemorrhage  30 min  Uncontrolled hemorrhage:  30 sec | Controlled hemorrhage:  60% of total  blood volume | Laparotomy+ packing | If MAP <25mmHg during blood withdrawal:  165ml/min of 0.9% saline solution  After shock (controlled hemorrhage) period:  Haemorrhage volume in a 3:1 ratio of 0.9% saline solution  Immediately after liver packing:  FFP and freeze-dried plasma: 60% of the volume of withdrawn blood  Spray-dried plasma: 33% of the volume of withdrawn blood | 0% | 33°C | intraperitoneal lavage after resuscitation/ no rewarming | Total  6.5 hours  hypothermia  4.5 hours | Hypothermia as part of the lethal triad- effects of spray-dried plasma on coagulation |
| Schnuringer B et al. 2011 ([36](#_ENREF_36)) | - Development of new model - Mortality | - n=20 | - Volume-contr. hem., via ven. cath, - Uncontr. hem. (grade IV liver injury or two grids with lacerations) | Directly after controlled haemorrhage and 15 min after liver injury | Controlled hemorrhage:  35% of total blood volume | Laparotomy + packing | After controlled hemorrhage:  1000ml lactated Ringer´s for MAP >60mmHg  After uncontrolled hemorrhage:  Lactated Ringer´s for MAP >60mmHg (max. 3500ml) | 30% | 35°C | Infusion of crystalloids for resus. after controlled hem. and abdominal cool packs after laparotomy/ rewarming with warm infusions for resus. after uncontrolled hem. | Total:  90-120min intubated, until 48 hours awake  Hypothermia:  ≈100min | Hypothermia as part of the lethal triad- standardization of a model |
| Bochicchio G et al. 2009 ([35](#_ENREF_35)) | - Blood loss - Time to hemostasis | - Modified chitosan: n=11 - Standard packing: n=7 | - Induction of isovolemic exchange transfusion with 60% of total blood volume - Uncontr. hem (grade V liver injury) | 30 sec | With modified chitosan: ≈ 810ml  Standard packing: ≈ 2200ml | Laparotomy | Hextend at a rate of 150ml/min until mean MAP 80% of preinjury value | - With modified chitosan: 0%   Standard packing: 100% | 32°C | External cooling and peritoneal lavage before trauma induction/ partial rewarming with warm infusions during resuscitation | Total:  1 hour  Hypothermia:  1 hour | Hypothermia as part of the lethal triad- effects on coagulation |
| Ding W et al. 2009 and 2010 ([17](#_ENREF_17), [18](#_ENREF_18)) | Ding 2009   - Mortality   Ding 2010:   - Mortality | - Control group (no resus.): n=6 (2009) n=8 (2010) - Primary anastomosis of SMA: n=6 (2009) n=8 (2010) - Temporary shunt of SMA:   n=6 (2009) n=8 (2010) | - Splenectomy - Transection of superior mesenteric artery (SMA) - Volume-conr. hem. via arterial cath. - Uncontr. hem. (grade V liver injury) | Controlled hemorrhage:  30 minutes  Uncontrolled hemorrhage:  30 sec | Controlled hemorrhage::  40 or 45% of total blood volume | Laparotomy | After splenectomy:  Warm lactated Ringer´s 3x the organ´s weight  Limited resuscitation:  Until MAP 60mmHg  After surgical repair:  Retransfusion of shed blood  Over entire study period:  Epinephrine, if MAP <40mmHg | Control group (no resus.):  100%  Primary anastomosis of SMA:  50%  Temporary shunt of SMA:  25% | 34.7°C | Infusion of 4°C lactated Ringer´s solution and intraperitoneal lavage with 4°C lactated Ringer´s solution/ rewarming with 38°C lactated Ringer´s solution | Total:  ≈7.5-8 hours  Hypothermia:  30 minutes | Hypothermia as part of the lethal triad- effects during hemorrhage |
| Hamilton GJ et al. 2011 ([20](#_ENREF_20)) | - Inflammatory response - Mortality | - n=30 | - Open femoral Fx by captive bolt gun - Vol. contr. hem. | Controlled hemorrhage:  30 minutes | Controlled hemorrhage:  60% of total blood volume | Laparotomy+ packing | Pre-hospital resuscitation:  3x volume of blood loss  Clinical resuscitation:  Lyophilized plasma equivalent to volume of contr. hem. | 0% | 33°C | Intraperitoneal lavage with cold saline during contr. hemorrhage/ active rewarming | Total:  ≈7 hours  Hypothermia:  ≈60 minutes | Hypothermia as part of the lethal triad- effects during hemorrhage and transfusion |
| Martini WZ et al. 2005 ([34](#_ENREF_34)) | - Coagulation profile | - n=24 | - Incision of the spleen (3mm) | No intervention | Measuring of bleeding cessation time | Laparotomy | Not available | 0% | 32°C | Before trauma induction by water-pumped blanket/ no rewarming | Total:  ≈3 hours  Hypothermia:  ≈2 hours | Hypothermia as part of the lethal triad- effects on coagulation |
| Holcomb JB et al. 1999 and Klemcke HG et al. 2005 ([21](#_ENREF_21), [25](#_ENREF_25)) | Holcomb JB:   - Blood loss - Mortality   Klemcke HG:   - In vitro coagulation - Blood loss - Mortality | Holcomb JB:   - n=19   Klemcke HG:   - Control: n=18 - rFVIIa 180µg/kg: n=18 - rFVIIa 720µg/kg: n=18 | - Splenectomy - 50% isovolemic blood exchange - Uncontr. hem. (grade V liver injury) | Uncontr. hem.:  30 sec | - Isovolemic blood exchange: 50% of total blood volume | Laparotomy and hepatic packing | - After splenectomy: 3x weight of the spleen lactated Ringers - Isovolemic blood exchange: Exchange of withdrawn blood with Hetatstarch - ≈4.5 min after hepatic injury: lactated Ringers at 260ml/min (MAP independent) - ≈4.5 min after hepatic injury: MAP dependent resuscitation with lactated Ringers | - Control: 66.6% - rFVIIa 180µg/kg: 55.6% - rFVIIa 720µg/kg: 66.6% | 32.5°C | Infusion of iv cold fluid during isovolemic blood exchange and resuscitation/ no rewarming | Total:  4 hours  Hypothermia:  ≈4 hours | Hypothermia as part of the lethal triad- effects on coagulation |
| Pursifull NF et al. 2006 ([28](#_ENREF_28)) | - Blood loss - Mortality | - Control group: n=5 - Experimental froup (FloSeal): n=5 | - Splenectomy and contralateral nephrectomy - Uncontr. hem. (grade V renal injury) | - Uncontr. hem.10 sec | - Control group (gelatine sponge): 540ml - Experimental group (FloSeal): 540ml | Celiotomy | - Splenectomy:3x the weight of the spleen lactated Ringers | Not available | 32°C | Before grade V renal injury by cooling blanket and intraperitoneal lavage with cold fluid/ rewarming 5min after hemostasis by warming blanket and Bair Hugger | Total:  ≈4 hours intubated, 10 days awake  Hypothermia:  ≈2 hours | Hypothermia as part of the lethal triad- effects on coagulation |
| Kheirabadi BS et al. 2013 ([24](#_ENREF_24)) | - Blood loss - Mortality | - Combat Ready Clamp (CRC) and control group without CRC: n=6 | - Splenectomy - 50% isovolemic blood exchange - Injury of femoral artery | - Uncontr. hem.15 sec | - Combat Ready Clamp (CRC): 5.25ml/kg - Control group without CRC: 75.8 ml/kg | Laparatomy | - Splenectomy:3x the weight of the spleen lactated Ringers - Isovolemic blood exchange: Exchange of withdrawn blood with Hetatstarch - Limited resuscitation: Hextent to maintain MAP >65mmHg | - Combat Ready Clamp (CRC): 0% - Control group without CRC: 83.3% | 34.5°C | During isovolemic blood exchange and with cooling blanket/ no rewarming | Total:  ≈3 hours intubated Hypothermia:  ≈2.5 hours | Hypothermia as part of the lethal triad- effects on coagulation |
| Kheirabadi BS et al. 2010 ([55](#_ENREF_55), [56](#_ENREF_56)) | - Blood loss - Mortality | - Regular gauze:   n=12   - Combat gauze:   n=15   - WoundStat: n=15 - FAST: n=13 | - Splenectomy - 50% isovolemic blood exchange - Injury of femoral artery | - Uncontr. hem.30 sec | - Regular gauze: 76.6ml/kg - Combat gauze: 55ml/kg - WoundStat: 67.9 ml/kg - FAST: 29.6ml/kg | Laparatomy | - Splenectomy:3x the weight of the spleen lactated Ringers - Isovolemic blood exchange: Exchange of withdrawn blood with Hetatstarch - Limited resuscitation: 500ml Hextent to raise MAP >65mmHg | - Regulat gauze: 91.7% - Combat gauze: 60% - WoundStat: 86.7% - FAST: 23% | 33°C | During isovolemic blood by infusion exchange and with cooling blanket/no rewarming | Total:  ≈4 hours intubated Hypothermia:  ≈2 hours | Hypothermia as part of the lethal triad- effects on coagulation |
| Sena MJ et al. 2013 ([29](#_ENREF_29)) | - Blood loss - Mortality | - Plain gauze pad: n=8 - Combat gauze pad: n=8 | - Splenectomy - 60% isovolemic blood exchange - Uncontr. hem. (grade V liver injury) | Uncontr. hem.:  30 sec | - Isovolemic blood exchange: 60% of total blood volume - Uncontrolled hemorrhage: Plain gauze pad (PG): 58ml/kg; Combat gauze pad (CG): 25ml/kg | Laparotomy and hepatic packing | - After splenectomy: 3x weight of the spleen lactated Ringers - Isovolemic blood exchange: Exchange of withdrawn blood with Hetatstarch - For 60 min after hepatic injury: warmed hextend for MAP of 80% of preinjury value | - PG: 50% - CG:12% | 32.5°C | Infusion of iv room temperature fluid during isovolemic blood exchange, external cooling and intraabdominal ice-packs/ partial rewarming with warm fluids during resuscitation | Total:  ≈2 hours after  Hypothermia:  ≈3 hours | Hypothermia as part of the lethal triad- effects on coagulation |
| Wang P et al. 2013 ([66](#_ENREF_66)) | - Mortality | - Control group: n=10 - Primary anastomosis: n=10 - Damage Control: n=10 | - Penetrating abdominal gun shot wound - Pressure-conr. hem. via arterial cath. | Controlled hemorrhage:  40 minutes | Controlled hemorrhage::  Not available | Laparotomy | Prehospital resuscitation:  Lactated Ringers until MAP 60mmHg  After surgical repair:  Retransfusion of shed blood  Over entire study period:  Epinephrine, if MAP <40mmHg | Control group (no resus.):  100%  Primary anastomosis:  50%  Damage Control:  30% | 34.6°C | Infusion of 4°C lactated Ringer´s solution during hemorrhagic shock/ rewarming during transfusion of shed blood by airway heating and humidification | Total:  ≈29-31 hours  Hypothermia:  ≈4-6 hours | Hypothermia as part of the lethal triad- effects during hemorrhage |
| Schreiber MA et al. 2002 ([67](#_ENREF_67)) | - Blood loss - Mortality - In vitro coagulation | - Control: n=10 - rFVIIa 180µg/kg: n=10   rFVIIa 720µg/kg: n=10 | - Splenectomy - 60% isovolemic blood exchange - Uncontr. hem. (grade V liver injury) | Uncontr. hem.:  30 sec | - Isovolemic blood exchange: 60% of total blood volume - Uncontrolled hemorrhage: Control: 21.87ml rFVIIa 180µg/kg: 1085ml - rFVIIa 720µg/kg: 1086ml | Laparotomy and hepatic packing | - After splenectomy: 3x weight of the spleen lactated Ringers - Isovolemic blood exchange: Exchange of withdrawn blood with albumin - After hepatic injury: Lactated Ringers until baseline MAP | - Control: 40% - rFVIIa 180µg/kg: 30% - rFVIIa 720µg/kg: 20% | 33°C | Infusion of iv room temperature fluid during isovolemic blood exchange and resuscitation, intraabdominal lavage/ no rewarming | Total:  ≈2.5 hours  Hypothermia:  ≈2 hours | Hypothermia as part of the lethal triad- effects on coagulation |
| Delgado AV et al. 2008 ([68](#_ENREF_68)) | - Blood loss - Coagulation profile Mortality | - HP: n=8 - FP: n=9 - PP: n=9 | - 60% isovolemic blood exchange - Uncontr. hem. (grade V liver injury) | - Uncontr. hem.: 30 sec | - Laparotomy sponges (LS): 4754ml - Fibrin patch (FP): 1213ml - Placebo patch (PP): 5495 | Laparotomy and hepatic packing | - Isovolemic blood exchange: Exchange of withdrawn blood with hextend - After hepatic injury: Hextend for 80% of baseline MAP | - HP: 87% - FP: 11% - PP: 100% | 32°C | Infusion of iv room temperature fluid during isovolemic blood exchange and external cooling/ partial rewarming with warm hextend during resus | Total:  ≈2.5 hours  Hypothermia:  ≈2 hours | Hypothermia as part of the lethal triad- effects on coagulation |
| Alam HB et al. 2009 ([69](#_ENREF_69)) | - Coagulation profile - Mortality | - Fresh whole blood: n=6 - Hextend: n=6 - FFP and RBC: n=6 - FFP: n=6 | - Femoral fx with capative bolt gun - Vol. control. Hem. - Uncontr. hem. (grade V liver injury) | - Controlled hemorrhage: 30 min - Uncontr. hemorrhage: 30 sec | - Contr. hem: 60% of total blood volume - Uncontr. hem: 400-600ml | Laparotomy + packing | - Contr. hem: 3x shed blood with normal saline (165ml/min) - Uncontr. hem: transfusions equal to volume withdrawn blood (50ml/min) | - Fresh whole blood (FWB): 0% - Hextend: 85% - FFP and RBC: 0% - FFP alone: 0% | 33°C | Infusion of room temperature fluid during resuscitation and intraperitoneal lavage/ partial rewarming with warmed transfusions | Total:  ≈7 hours  Hypothermia:  ≈ 4 hours | Hypothermia as part of the lethal triad- effects on coagulation |
