# Supplementary material for: Relevance of induced and accidental hypothermia after trauma-haemorrhage–what do we know from experimental models in pigs?
Source: Intensive Care Med Exp. 2014 May 15;2:16. doi: 10.1186/2197-425X-2-16 (PMC4512998; doi:10.1186/2197-425X-2-16)
Supplement: Supplementary file 2 — Additional file 2: Table S2: Induced hypothermia [10, 11, 30, 40, 45, 47–50]. (DOCX 152 KB) [file 40635_2013_17_MOESM2_ESM.docx]

| Author | Endpoints | Number of animals | Insult | Time to resuscitation | Blood loss | Surgical procedure | Resuscitation | Mortality | Hypo-thermia | Hypothermia induction/  rewarming | Study period/  hypothermia period | Thesis |
| --- | --- | --- | --- | --- | --- | --- | --- | --- | --- | --- | --- | --- |
| Mohr J et al. 2013 ([40](#_ENREF_40)) | - Coagulation profile | - n=40 | - Pulmonary contusion via capative bolt gun - Liver incision - Controlled hemorrhage via arterial line | 90 min | 30±5mmHg (max. of 45% of total blood volume | Midline laparotomy and liver packing | 3 times the shed blood volume (colloids and crystalloids used in a relation of 1:8) | 0% | 34°C | Venous catheter (CoolGard) after resuscitation/ rewarming via venous catheter (CoolGard) | total:  16.5 intubated hypothermia:  3 hours | Effects of therapeutically induced hypothermia on coagulation |
| Sailhamer EA et al. 2007 ([10](#_ENREF_10)) | - Mortality - Infections - Neurologic status - Organ function - Coagulation profile | - Vascular injury: n=10 - Vascular + colon injury: n=8 - Vascular + colon + splenic injury: n=8 | - Uncontrolled hemorrhage by iliac artery and vein injury - Aortic laceration - Colon injury - Splenic injury | ≈ 35 min | MAP <20mmHg  (≈ 50% of total blood volume) | - Thoracotomy - Abdominal incision | Autotransfusion of shed blood volume | Hypothermia:   - 10% (vascular injury) - 12.5% (vascular + colon injury) - 25% (vascular + colon + splenic injury)   Normothermia:   - 100% | 10°C | 5 min after aortic laceration with roller pump and heat exchanger/rewarming with roller pump and heat exchanger | total:  5 hours intubated, 6 weeks awake hypothermia  60 min | Effects of therapeutically induced hypothermia on bleeding, infections and survival |
| Takasu A et al. 2000 ([47](#_ENREF_47)) | - Cooling time - Mortality | - Normothermia: n=6 - Surface cooling: n=6 | - Lethal, controlled hemorrhage via venous line | No resuscitation, termination at 4h | 30ml/kg BW | None | No resuscitation | Normothermia:  66%  Surface Cooling:  100% | 35.3°C | 5 min after end of bleeding by ice packs and evaporative cooling/no rewarming | total:  108-175 minutes hypothermia  ≈90 minutes | Effects of therapeutically induced surface cooling on survival |
| Norio H et al. 2002 ([45](#_ENREF_45)) | - Cooling time - Mortality | - Normothermia: n=7 - Hypothermia: n=7 | - Controlled hemorrhage via venous line - Uncontrolled hemorrhage (aortotomy) | 25 min | 25ml/kg BW | Celiotomy | Isolated infusion of 500ml lactated Ringer´s solution | Normothermia:  85.7%  Hypothermia:  28.6% | 35.5°C | Directly after aortotomy/ no rewarming, animals left at room temperature | total:  Max. 4 hours (mean: 220 minutes) hypothermia  20 min, then left at room temperature | Effects of therapeutically induced hypothermia on survival |
| Alam HB et al. 2004, Alam HB et al. 2006, Alam HB et al. 2008 ([11](#_ENREF_11), [51](#_ENREF_51), [52](#_ENREF_52)) | Alam HB 2004:   - Organ function - Mortality   Alam HB 2006:   - Organ function - Mortality - Neurologic function   Alam HB 2008:   - Mortality - Organ function - Neurologic function | Alam HB 2004:   - Normothermia: n=8 - Hypothermia:   Slow (0.5°C/  min): n=8  Medium (1°C/  min): n=8  Fast (2°C/  min): n=8  Alam HB 2006:   - Normothermia: n=10 - Rewarming:   Slow (0.25°C/  min): n=10  Medium (0.5°C/  min): n=10  Fast (1°C/min):  n=10  Alam HB 2008:   - Hypothermia 60 min: n=10 - Hypothermia 120 min: n=12 | - Uncontrolled hemorrhage   (aortotomy, laceration of iliac vessels) | ≈ 90 min | ≈ 50% of total blood volume | - Thoracotomy - Lower abdominal incision | Autotransfusion of shed blood and of whole blood | Normothermia:  100%  Hypothermia:  Slow (0.5°C/min): 62.5%  Medium (1°C/min): 37.5%  Fast (2°C/min): 12,5%  ([11](#_ENREF_11))  Hypothermia 60 min: 8%  Hypothermia 120 min: 50%  ([52](#_ENREF_52))  Rewarming:  Slow (0.25°C/min): 50%  Medium (0.5°X/min): 10%  Fast (1°C/min): 70%  ([51](#_ENREF_51)) | 10° C | After 5min of aortic lesion with roller pump and heat exchanger via aortic catheter/ rewarming with roller pump and heat exchanger | total:  6 hours intubated, 6 weeks awake hypothermia  60 (or 120)min | Effects of therapeutically induced hypothermia on organ function, inflammation and survival |
| Wu X et al. 2005 ([48](#_ENREF_48)) | - Mortality | - Normothermia: n=8 - Surface cooling: n=8 - Cold infusion and surface cooling: n=8 | - Controlled hemorrhage via ??? - Splenectomy (at 35 min) | > 40 min | - 68.6ml/kg/h for 0-35 min - 20.0ml/kg/h for 35-95 min - 10.0ml/kg/h for 95-180 min | Laparotomy | - Between 40-180 min:   0.9% saline solution when MAP <90 mmHg   - 180 min:   Shed blood from first 30 min, additional lactated Ringer´s solutuin until MAP >70mmHg | - Normothermia:   75%   - Surface cooling:   25%   - Cold infusion and surface cooling:   50% | 34°C | 40 min after induction of hem. by cold infusion and surface cooling/ rewarming | total:  24 hours hypothermia:  ≈ 11.5 hours | Effects of therapeutically induced hypothermia on survival |
| Wladis A et al. 2001 ([30](#_ENREF_30)) | - Mortality - Hemodynamic response | - Hypothermia: n=9 - Normothermia: n=11 | - Controlled hemorrhage | No resuscitation | 50% of total blood volume | none | none | Normothermia:  12.5%  Hypothermia:  0% | 30°C | After hemorrhage with hypothermic bed/no rewarming, animals left at room temperature | total:  4 hours hypothermia  ≈4 hours | Effects of therapeutically induced hypothermia on metabolic function |
| Takasu A et al. 2003 ([41](#_ENREF_41)) | - Mortality - Oxygen metabolism | - Hypothermia: n=5 - Normothermia: n=5 | - Volume-controlled hemorrhage | No resuscitation | 30ml/kg BW | none | none | - Hypothermia:   0%   - Normothermia:   60-100% | 34°C | Arterio-venous cooling before the end of hemorrhage//no re-warming procedure (an left at room temperature) | total:  Max. 4 hours hypothermia  20 min, then animals left at room temperature | Effects of therapeutically induced hypothermia on short-term survival |
| Iyegha UP et al. 2012 ([42](#_ENREF_42)) | - Mortality - Markers of organ injury - Neurologic function | - Normothermia: n=7 - Hypothermia: n=9 | - Chest trauma by captive bolt gun (multiple rip fracture with underlying pulmonary contusion) - Pressure contr. hem. - Uncontr. hem. (grade III liver injury) | 60 min | Controlled hemorrhage: MAP ≈55mmHg | Laparotomy | Limited resuscitation for 1h: Lactated Ringer´s for SBP >80mmHg  Full resuscitation: Lactated Ringer´s for SBP >90mmHg and transfusion of shed blood for Hb >6g/dl | Normothermia: 29%  Hypothermia 11% | 34°C | Ice packs before trauma induction/ within the resuscitation process with warming blankets | total:  24 hours intubated and 24 hours awake hypothermia  ≈2.5 hours | Effects of therapeutically induced hypothermia on organ function |
| *George ME et al. 2010 (*[*46*](#_ENREF_46)*)* | - Mortality - Markers of cellular stress and organ dysfunction | - Normothermia: n=5 - Mild hypothermia: n=7 - Severe hypothermia: n=7 | Pressure-controlled, via venous catheter | 45 min | SAP 45-55mmHg | None | Limited resuscitation: Hextend for SBP >80mmHg  Full resuscitation:  for SBP >90mmHg Hextend (max. 500ml) then Ringer´s solution  for Hb >6gm/dl transfusion of shed blood | Normothermia: 60%  Mild hypothermia: 20%  Severe hypothermia: 0% | Mild: 36°C  Severe:  33°C | Ice packs within limited resuscitation/ active rewarming before full resuscitation | Total:  24 hours intubated, 24 hours awake  Hypothermia:  ≈7 hours | *Effects of therapeutically induced hypothermia on survival and organ function* |
| Groger M et al. 2013 ([43](#_ENREF_43)) | - Organ function/damage - Coagulation profile | - 32°C: n=7 - 35°C: n=7 - 38°C: n=6 | - Volume contr. hem. | - 4 hours | Controlled hemorrhage:  40% of total blood volume, thereafter MAP 30±3mmHg | Laparotomy | Shed blood, Ringer´s solution and hydroxyethyl starch:  -10ml/kg hr each  - 10ml/kg hr each if CVP or PAOP >18mmHg, titrated to maintain ITBV at 25-30ml/kg  - norepinephrine for baseline MAP (but HR <160/min) | 32°C  ≈10%  35°C  ≈10%  38°C  ≈30% | 32°C or  35°C | Before hemorrhage by external heat exchanger/ rewarming by external heat exchanger | Total:  26 hours  Hypothermia:  16 hours | Effects of therapeutically induced hypothermia on survival and organ function, inflammation and coagulation |
| Wladis A et al. 1998 ([49](#_ENREF_49)) | - Metabolic and endocrine parameters - Mortality | - Normothermia: n=8 - Hypothermia: n=9 | - High-energy gun shot wound of hind leg - Controlled hemorrhage | No resuscitation | 50% of total blood volume | none | none | Normothermia:  14.2%  Hypothermia:  0% | 30°C | After hemorrhage with hypothermic bed/no rewarming, animals left at room temperature | total:  ≈4.5 hours hypothermia  ≈3 hours | Effects of therapeutically induced hypothermia on metabolic function |
